# Validated Stability-Indicating HPLC-DAD Method for the Simultaneous Determination of Diclofenac Sodium and Diflunisal in Their Combined Dosage Form

**DOI:** 10.3797/scipharm.1301-24

**Published:** 2013-03-17

**Authors:** Rasha A. Shaalan, Tarek S. Belal

**Affiliations:** Pharmaceutical Analytical Chemistry Department, Faculty of Pharmacy, University of Alexandria, Elmessalah 21521, Alexandria, Egypt.

**Keywords:** Diclofenac sodium, Diflunisal, HPLC-DAD, Stability-indicating determination, Forced degradation, Suppositories

## Abstract

A simple, rapid, and highly selective HPLC-DAD method was developed for the simultaneous determination of diclofenac sodium (DIC) and diflunisal (DIF) in pure form and in their combined formulation. Effective chromatographic separation was achieved using a Zorbax SB-C8 (4.6×250 mm, 5 μm particle size) column with a mobile phase composed of 0.05 M phosphoric acid, acetonitrile, and methanol in the ratio of 40:48:12 (by volume). The mobile phase was pumped isocratically at a flow rate of 1 mL/min, and quantification of the analytes was based on measuring their peak areas at 228 nm. The retention times for diflunisal and diclofenac were about 7.9 and 9.5 min, respectively. The reliability and analytical performance of the proposed HPLC procedure were statistically validated with respect to system suitability, linearity, ranges, precision, accuracy, specificity, robustness, detection, and quantification limits. Calibration curves were linear in the ranges of 5–100 μg/mL for both drugs with correlation coefficients >0.9998. The proposed method proved to be selective and stability-indicating by the resolution of the two analytes from four of their related substances and potential impurities as well as from forced-degradation (hydrolysis, oxidation, photolysis, and dry heat) products. The validated HPLC method was successfully applied to the analysis of DIC and DIF in their combined dosage form (suppositories). The proposed method made use of the diode array detector (DAD) as a tool for peak identity and purity confirmation.

## Introduction

**Fig. 1:**
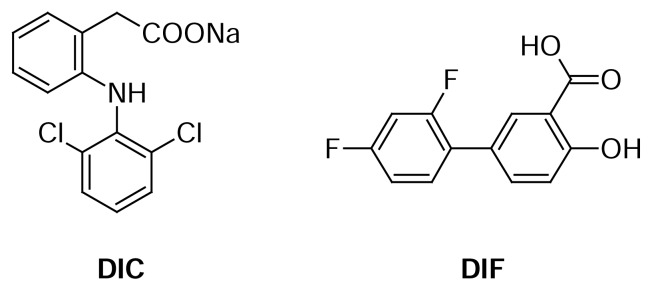
Chemical structures of diclofenac sodium (DIC) and diflunisal (DIF).

Diclofenac sodium (DIC) (Fig. 1), chemically known as sodium {2-[(2,6-dichlorophenyl)-amino]phenyl}acetate [1], is a phenylacetic acid derivative non-steroidal anti-inflammatory drug (NSAID). It is used for the relief of pain and inflammation in various conditions: musculoskeletal and joint disorders such as rheumatoid arthritis and osteoarthritis, soft tissue disorders such as sprains, and other painful conditions such as renal colic, acute gout, dysmenorrhoea, migraine, and after some surgical procedures [2]. Both the British Pharmacopoeia (BP) and the United States Pharmacopeia (USP) describe a potentiometric non-aqueous titration procedure for the assay of DIC bulk form and HPLC methods for the assay of various DIC formulations [1, 3], however the prolonged-release capsules are assayed using A_max_ measurement at 275 nm in the BP [1]. Moreover, the quantification of DIC in its various drug formulations and/or biological samples was addressed in many reports. Liquid chromatography using various detection modes has been widely applied. Examples of these reports are HPLC with UV detection [4–6], HPLC with electrochemical detection [7], and HPLC with mass spectrometric detection [8, 9]. In addition, other analytical techniques involved the use of potentiometric membrane sensors [10], cyclic and differential-pulse voltammetry [11], spectrophotometry [12, 13], chemometric spectrophotometry [14], spectrofluorimetry [15], infrared and Raman spectroscopy [16], gravimetry [17], GC-MS [18], HPTLC [19], and capillary zone electrophoresis [20].

Diflunisal (DIF) (Fig. 1), chemically known as 2′,4′-difluoro-4-hydroxybiphenyl-3-carboxylic acid [1], is a salicylic acid derivative NSAID. Diflunisal is given in the acute or long-term management of mild to moderate pain, and inflammation associated with rheumatoid arthritis and osteoarthritis [2]. The official monograph of DIF in the BP describes a titrimetric procedure and a spectrophotometric A_max_ method at 315 nm for the analysis of the powder form and tablets, respectively [1], on the other hand, HPLC has been recommended for the assay of both DIF powder and tablets in the USP [3]. Several methods have been reported describing the determination of DIF in different matrices. These methods expose the application of an ion-selective electrode [21], differential-pulse polarography [22], differential-pulse and square-wave stripping voltammetry [22, 23], derivative spectrophotometry [24], chemometric spectrophotometry [25], and synchronous fluorescence spectrometry [26, 27]. Also, the scientific literature showed the use of separation techniques such as HPLC-UV detection [25], HPLC-fluorescence detection [28], TLC-densitometry [29], and capillary electrophoresis with luminescence detection [30]. Finally, the determination of several NSAIDs including DIC and DIF in water samples was carried out using liquid chromatography coupled with diode array detection (LC-DAD) [31] and LC-DAD-MS [32].

Diclofenac sodium (DIC) and diflunisal (DIF) are co-formulated in suppository dosage form [33] directed to be used in inflammatory and painful conditions such as rheumatoid arthritis, osteoarthritis, postoperative conditions, acute gouty attack, renal and biliary colic, and dysmenorrhoea. Reviewing the literature revealed that only one report described the simultaneous determination of DIC and DIF using derivative and ratio-spectra derivative spectrophotometry, TLC-densitometry, and HPLC [34]. Nothing was mentioned in this previous report about the forced degradation behavior of the two analytes or the resolution of the analytes from their related substances, so consequently, none of these procedures can be considered stability-indicating.

The aim of the present study is the development, validation, and application of a simple, reliable, and specific HPLC-DAD method for the analysis of the DIC-DIF drug combination. The method was thoroughly tested for its specificity and stability-indicating properties by the resolution of both drugs from four of their related substances: 2,6-dichloroaniline (DCA), 2,6-dichloro-*N*-phenylaniline (PDCA), 2-chloro-*N*-(2,6-dichlorophenyl)-*N*-phenylacetamide (DCPCA), and biphenyl-4-ol (BPL) [1, 35, 36] as well as from their forced hydrolytic, oxidative, dry heat, and photolytic degradation products.

## Experimental

### Instrumentation

The HPLC-DAD system consisted of the Agilent 1200 series (Agilent Technologies, Santa Clara, CA, USA) (quaternary pump, vacuum degasser and diode array, and multiple wavelength detector G1315 C/D and G1365 C/D) connected to a computer loaded with Agilent ChemStation Software. A Rheodyne manual injector with a 20 μL loop was used. The column used was the Zorbax SB-C8 (4.6 × 250 mm, 5 μm particle size) (Agilent).

### Materials

Diclofenac sodium (DIC) and diflunisal (DIF) were kindly supplied by Alexandria Company for Pharmaceuticals, Alexandria, Egypt. The two related substances 2,6-dichloro-*N*-phenylaniline (PDCA) and 2-chloro-*N*-(2,6-dichlorophenyl)-*N*-phenylacetamide (DCPCA) were kindly donated by Pharco Pharmaceuticals Company, Alexandria, Egypt. Biphenyl-4-ol (Merck Schuchardt OHG, Hohenbrunn, Germany), 2,6-dichloroaniline (Sigma-Aldrich, St. Louis, MO, USA), HPLC-grade acetonitrile (Scharlau Chemie S.A., Sentmenat, Spain), HPLC-grade methanol (Sigma-aldrich Chemie GmbH, Buchs, Switzerland), analytical grade of orthophosphoric acid, hydrochloric acid, sodium hydroxide, 30% hydrogen peroxide, and high-purity distilled water were used. The pharmaceutical preparation assayed in the study is Rheumafen Forte® suppositories (Glaxo Wellcome Egypt S.A.E., El-Salam City, Cairo, Egypt, BN 082345A) labeled to contain 100 mg diclofenac sodium and 200 mg diflunisal per suppository.

### General procedure

The mobile phase was prepared by mixing 0.05 M orthophosphoric acid, acetonitrile, and methanol in the ratio of 40:48:12 (by volume). The mobile phase was filtered by passing through a 0.45 μm pore size membrane filter prior to use. The flow rate was 1.0 mL/min. The injection volume was 20 μL. The eluant was monitored by the diode array detector from 190 to 400 nm, and chromatograms were recorded at 228 nm. All determinations were performed at 25°C.

DIC stock solution (1000 μg/mL) and DIF stock solution (1000 μg/mL) were prepared in HPLC-grade methanol. The working solutions were prepared by dilution of the stock solutions with HPLC-grade acetonitrile to reach the concentration range 5–100 μg/mL for both drugs. Triplicate injections were made for each concentration and chromatographed under the previously described LC conditions. The peak areas were plotted against the corresponding concentrations to construct the calibration graphs.

### Assay of the pharmaceutical dosage form

The active ingredients of one Rheumafen Forte® suppository were extracted into HPLC-grade methanol with the aid of sonication for 15 minutes. The methanolic extract was filtered and completed to volume (100 mL) with the same solvent. Aliquots of the dosage form extract were diluted with HPLC-grade acetonitrile to obtain final concentrations within the specified range and then treated as under the general procedure. Recovered concentrations were calculated from the corresponding calibration graphs. For the standard addition assay, sample solutions were spiked with aliquots of standard solutions of both compounds to obtain total concentrations within the previously specified ranges then treated as under the general procedure. Recovered concentrations were calculated by comparing the analyte response with the increment response attained after the addition of the standard.

### Forced degradation and stability-indicating study

A stock solution of each of the four related substances (1000 μg/mL) was prepared in HPLC-grade methanol. Aliquots of these stock solutions were added to the two drugs under analysis and the mixture was diluted to volume with HPLC-grade acetonitrile. This mixture was chromatographed under the previously described LC conditions. In addition, forced degradation studies were carried out on DIC and DIF standards according to the following conditions:

Acidic and basic conditions: DIC and DIF solutions were treated with 1 mL of 1 M HCl or 1 M NaOH. The solutions were placed in a water bath at 90°C for 2 hr, except for DIC solution in HCl which was kept in a water bath at 60°C for 30 min. After the specified time, all solutions were neutralized by adjusting the pH to 7.0 and then diluted to volume with HPLC-grade acetonitrile.

Oxidation with H_2_O_2_: DIC and DIF solutions were treated with 0.5 mL of hydrogen peroxide 5%. The solutions were placed in a water bath at 90°C for 1 hr. After the specified time intervals, the solutions were diluted to volume with HPLC-grade acetonitrile.

Photolytic degradation: An amount of each drug powder (100 mg) was subjected to UV irradiation at 254 nm for 48 hrs. After the specified time, each powder was dissolved in methanol, and aliquots of these methanolic stocks were diluted to volume with HPLC-grade acetonitrile.

Dry heat degradation: An amount of each drug powder (100 mg) was kept in an oven at 90° C for 7 hrs. After the specified time, each powder was dissolved in methanol, and aliquots of these methanolic stocks were diluted to volume with HPLC-grade acetonitrile.

After the previous treatments, solutions were filtered with a 0.45 μm filtration disk prior to injection to the column.

## Results and Discussion

### Optimization of chromatographic conditions

The reversed-phase HPLC assay of the DIC-DIF mixture was addressed in only one previous publication [34]. The published method involved the use of the Hypersil ODS column and an isocratic mobile phase composed of methanol, water, and acetic acid (80:20:0.01) [34]. This previous study did not demonstrate the selectivity of the method to allow the resolution of the two analytes from their related substances and forced-degradation products. To overcome this shortage, an isocratic liquid chromatographic method coupled with diode array detection was developed to provide a suitable procedure for the reliable selective stability-indicating and quality control analysis of the DIC and DIF mixture. The most important aspect in the LC method development is the achievement of sufficient resolution with acceptable peak symmetry in a reasonable analysis time. To achieve this goal, several experiments were carried out in order to optimize both the stationary and mobile phases. For the stationary phase, several reversed-phase columns (Zorbax SB-C8 (4.6 × 250 mm), Zorbax SB-C18 (4.6 × 250 mm), Zorbax Eclipse XDB-C18 (4.6 × 150 mm), and Waters Symmetry C18 (3.9 × 150 mm)) were tested. All these columns gave clear separation between the two analytes, however, successful resolution of the analytes from the tested related substances and degradation products was attained by using the Zorbax SB-C8 (4.6 × 250 mm), and hence it was used in this study.

Several mobile phase combinations were tested using various proportions of different aqueous phases and organic modifiers. The best resolution of the two analytes from the related substances and forced-degradation products within acceptable analysis time was obtained through an isocratic elution using a mobile phase consisting of 0.05 M phosphoric acid solution, acetonitrile, and methanol in the ratio 40:48:12 (by volume). Increasing the proportion of the aqueous phase led to prolonged retention times and broad peaks, whereas increasing the organic proportion led to inadequate separation of the peaks.

Diode array detection enhances the power of HPLC and is an elegant option for assessing method specificity by monitoring the recorded spectra during peak elution. Quantification was achieved using diode array detection based on peak area measurement. Both analytes exhibited considerable absorption all over the range 200–320 nm. DIC showed an absorption band with a maximum at 276 nm. On the other hand, DIF showed a characteristic broad absorption spectrum with a higher maximum at 228 and a lower one at 315 nm, in addition to an intermediate shoulder at 250 nm. The wavelength 228 nm was selected for quantification of the two analytes since it corresponds to a high absorption for both of them; in addition, it was found suitable for recording the forced-degradation chromatograms.

**Fig. 2:**
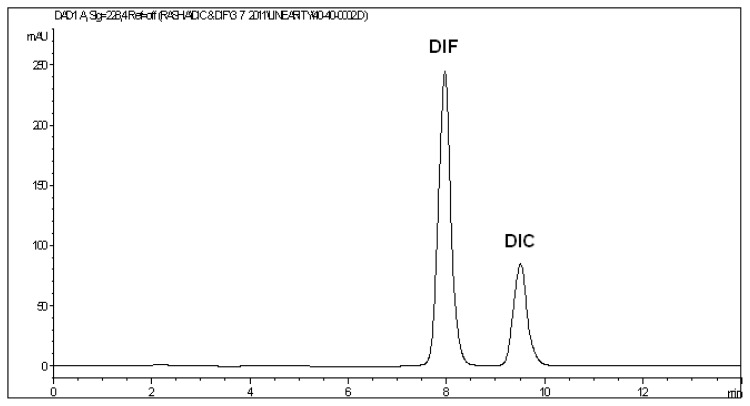
HPLC chromatogram of a mixture of 40 μg/mL DIC and 40 μg/mL DIF at 228 nm.

The above described chromatographic conditions showed symmetric peaks and adequate resolution between DIF and DIC. Fig. 2 shows a typical chromatogram for the separation of the two analytes. DIF and DIC eluted at retention times 7.93 ± 0.028 and 9.47 ± 0.033 min, respectively. A value of 1.5 for resolution implies a complete separation between two consecutive peaks [1]. Resolution (R_s_) and selectivity (α) for the mixture under analysis are 3.24 and 1.19, respectively. Finally, column performance (apparent efficiency) can be expressed by the number of theoretical plates (N) which equals 5080 and 5820 for DIF and DIC, respectively.

### Validation of the proposed method

#### Linearity and concentration ranges

The linearity of the proposed HPLC procedure was evaluated by analyzing a series of different concentrations (n=7) for each of the two analytes. The linear regression equations were generated by least squares treatment of the calibration data. Under the optimized conditions described above, the measured peak areas at 228 nm were found to be proportional to the concentrations of the analytes. Table 1 presents the performance data and statistical parameters including linear regression equations, concentration ranges, correlation coefficients, standard deviations of the intercept (S_a_), slope (S_b_), and standard deviations of residuals (S_y/x_). Regression analysis shows good linearity as indicated from the correlation coefficient values (>0.9998). In addition, the deviation around the slope can be further evaluated by calculation of the RSD% of the slope (S_b_%) which were found to be less than 1.0%. In addition to the previous parameters, linearity can be further guaranteed by the analysis of variance (ANOVA) test. The most important statistic in this test is the F-value which is the ratio of the mean of squares due to regression divided by the mean of squares due to residuals. High F-values reveal an increase in the mean of squares due to regression and a decrease in the mean of squares due to residuals. The greater the mean of squares due to regression, the steeper the regression line. The smaller the mean of squares due to residuals, the less the scatter of experimental points around the regression line. Consequently, regression lines with high F-values (low significance F) are much better than those with lower ones. Good regression lines show high values for both r and F statistical parameters [37, 38].

#### Detection and quantification limits

According to the pharmacopoeial recommendations [1, 3], the limit of detection is defined as the concentration that has a signal-to-noise ratio of 3:1, while for the limit of quantification, the ratio considered is 10:1. The LOD and LOQ values for DIC and DIF were calculated and are presented in Table 1.

#### Precision and accuracy

The within-day (intra-day) precision and accuracy for the proposed method were studied at three concentration levels for each compound using three replicate determinations for each concentration within one day. Similarly, the between-day (inter-day) precision and accuracy were tested by analyzing the same three concentrations for each compound using three replicate determinations repeated on three days. Recoveries were calculated using the corresponding regression equations and they were satisfactory. The percentage relative standard deviation (RSD %) and percentage relative error (E_r_ %) did not exceed 2.0% proving the high repeatability and accuracy of the developed method for the estimation of the analytes in their bulk form (Table 2).

#### Selectivity and specificity

Method selectivity was examined by preparing several laboratory-prepared mixtures of the two compounds at various concentrations within the linearity ranges mentioned in Table 1. These mixtures were of different ratios both above and below the normal ratio expected in the dosage form. The laboratory-prepared mixtures were analyzed according to the previously described procedure. The analysis results, including the percentage relative standard deviation (RSD %) and the percentage relative error (E_r_ %) values shown in Table 3, were satisfactory thus validating the selectivity, precision, and accuracy of the developed method and demonstrating its capability to resolve and quantify the analytes in different ratios. Specificity is defined as the ability to access unequivocally the analyte in the presence of components that may be expected to be present, such as impurities, degradation products, and matrix components [3], and this will be demonstrated in detail in the following sections of this study.

#### Robustness

Robustness was examined by evaluating the influence of small variations in different conditions such as the concentration of phosphoric acid solution (± 0.005 M), source of acetonitrile (Scharlau Chemie S.A., Spain or SDS, France or Labscan, Poland), source of methanol (Sigma-aldrich Chemie GmbH, Buchs, Switzerland or LabScan Analytical Sciences, Poland), working wavelength (± 2 nm), flow rate (± 0.05 mL/min), and column temperature (± 2°C). These variations did not have any significant effect on the measured responses or the chromatographic resolution. RSD% for the measured peak areas using these variations did not exceed 3%.

#### Stability of solutions

The stability of standard working solutions as well as sample solutions in the diluting solvent (HPLC-grade acetonitrile) was examined and no chromatographic changes were observed within 24 hours at room temperature. Also, the stock solutions prepared in HPLC-grade methanol were stable for at least two weeks when stored refrigerated at 4ºC. Retention times and peak areas of the drugs remained unchanged and no significant degradation was observed during these periods.

## Analysis of the pharmaceutical dosage form

**Fig. 3:**
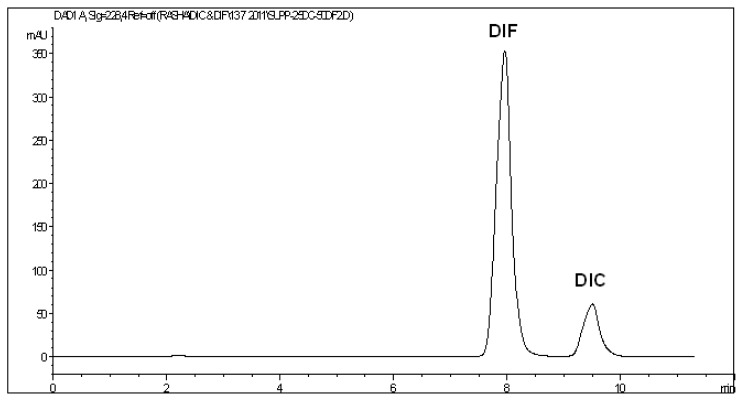
HPLC chromatogram of a mixture of 25 μg/mL DIC and 50 μg/mL DIF obtained from Rheumafen Forte® suppositories at 228 nm.

The optimized HPLC-DAD procedure was applied for the assay of this drug combination in the pharmaceutical formulation available in the local market (Rheumafen Forte® suppositories). The active ingredients were extracted with the same solvent used for the preparation of the standard stock solutions (HPLC-grade methanol), then dilution was made with HPLC-grade acetonitrile to reach concentration levels within the specified ranges. The active ingredients eluted at their specific retention times and no interfering peaks were observed from any of the inactive ingredients or the suppository base (Fig. 3). The diode array detection enabled peak purity verification where no signs of co-elution from any of the inactive components were detected. Recoveries were calculated using both external standard and standard addition methods. The assay results revealed satisfactory accuracy and precision as indicated from % recovery, SD, and RSD% values (Table 4). Furthermore, a reference RP-HPLC method [34] was applied for the estimation of DIC and DIF in their combined formulation. Recovery data obtained from the developed HPLC method were statistically compared with those of the reference method using the Student’s t- and the variance ratio F-tests. In both tests, the calculated values did not exceed the theoretical ones at the 95% confidence level, which indicated that there were no significant differences between the recoveries obtained from the developed method and those of the reference method (Table 4). It is evident from these results that the proposed method is applicable to the assay of this drug combination with a satisfactory level of selectivity, accuracy, and precision.

### Forced-degradation and stability-indicating aspects

**Fig. 4:**
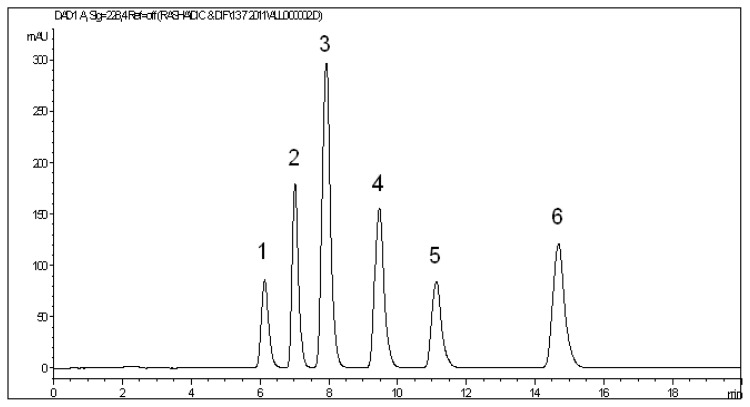
HPLC chromatogram of a mixture containing (1) biphenyl-4-ol (BPL), (2) 2,6-dichloroaniline (DCA), (3) diflunisal, (4) diclofenac, (5) 2-chloro-*N*-(2,6-dichlorophenyl)-*N*-phenylacetamide (DCPCA), and (6) 2,6-dichloro-*N*-phenylaniline (PDCA).

The optimized HPLC method was applied to test the chromatographic behavior of three of the related substances of diclofenac: 2,6-dichloroaniline (DCA), 2,6-dichloro-N-phenylaniline (PDCA), and 2-chloro-*N*-(2,6-dichlorophenyl)-*N*-phenylacetamide (DCPCA), in addition to the diflunisal-related substance: biphenyl-4-ol (BPL). The four related substances eluted at retention times 6.14, 7.03, 11.14, and 14.69 min for BPL, DCA, DCPCA, and PDCA, respectively. Fig. 4 illustrates the separation of a mixture containing the two parent compounds together with their related substances. Resolution (R_s_) is a measure of the degree of separation between adjacent peaks. Resolution was calculated between every two consecutive peaks, and it was found to be not less than 2.42 which revealed an excellent baseline separation between the eluted peaks.

Forced degradation experiments were carried out on DIC and DIF in order to produce the possible relevant degradation products and test their chromatographic behavior using the developed method. Hydrolytic, using strong basic (1 M NaOH) and strong acidic (1 M HCl) media, oxidative (5% H_2_O_2_), UV photolytic, and dry heat degradation experiments were conducted, and the resulting chromatograms were compared with those obtained from standard untreated solutions of the two compounds.

**Fig. 5:**
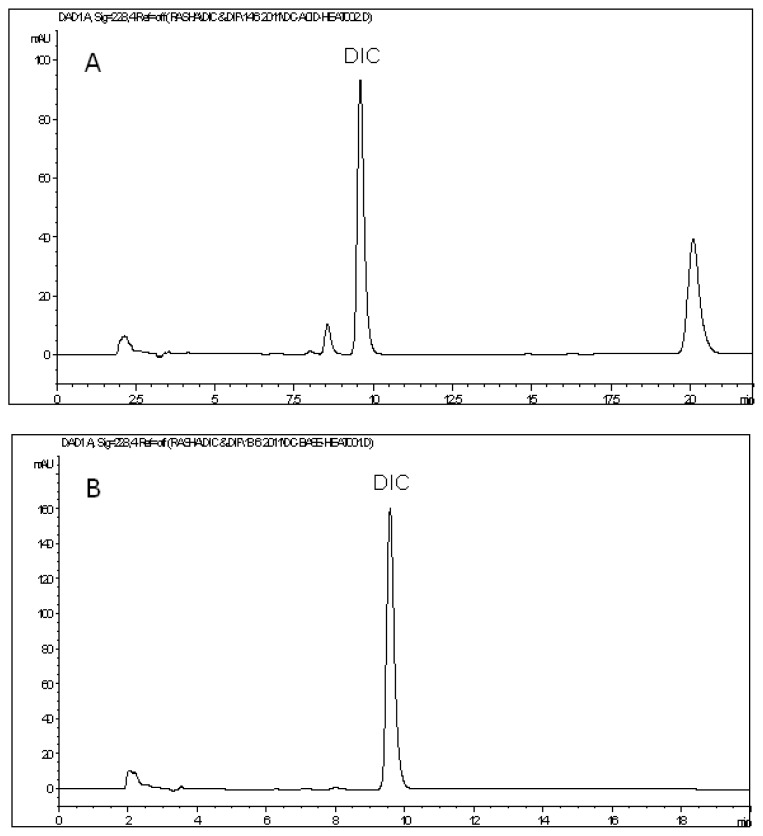

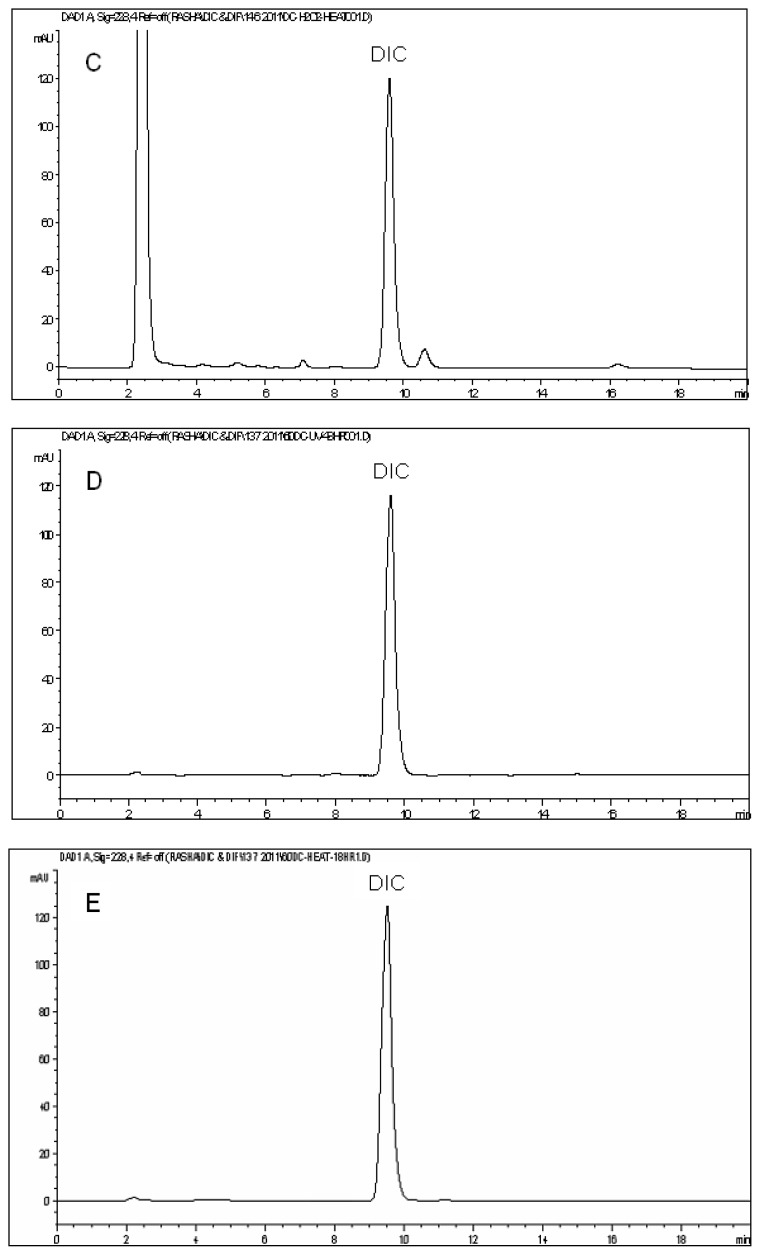
HPLC chromatograms of diclofenac sodium after exposure to acid degradation (A), base degradation (B), oxidative degradation (C), photolytic UV degradation (D), and thermal dry heat degradation (E).

DIC readily decomposes in acidic medium. Heating DIC with 1 M HCl at 60°C for 30 min revealed a remaining DIC peak which was about 53% of the expected area, in addition to the appearance of a minor degradation peak at 8.58 min and another major peak at 20.10 min (Fig. 5A). On the other hand, DIC appeared to be stable without any degradation in alkaline medium. Fig. 5B shows the peak of intact DIC after being heated at 90°C for 2 h with 1 M NaOH. Oxidative degradation with H_2_O_2_ at 90°C for 1 hr caused about a 20% reduction in the peak area of DIC. Several minor degradation peaks can be seen in the chromatogram at retention times 5.20, 7.06, 10.60, and 16.21 min (Fig. 5C). The degradation peak at 7.06 min shows an absorption spectrum that is very similar to that of the related substance, 2,6-dichloroaniline (DCA). This observation is in agreement with a previous work, which suggested that DCA is actually one of the oxidative degradation products of DIC [36]. Finally, no signs of degradation of DIC could be observed under UV photolytic and dry heat conditions. The DIC peak appeared at its specific retention time with an area almost identical to that of the standard at the same concentration, and additionally, the chromatograms did not show any extra peaks (Fig. 5D and 5E).

**Fig. 6:**
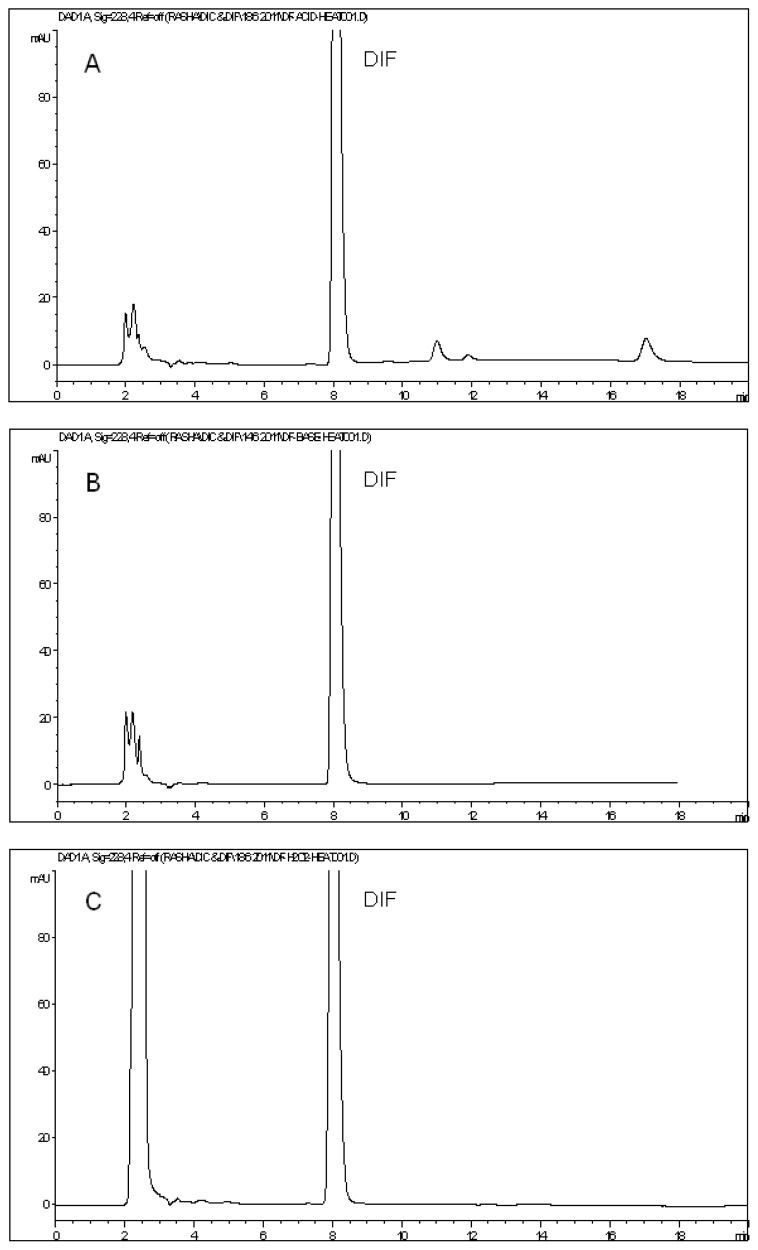

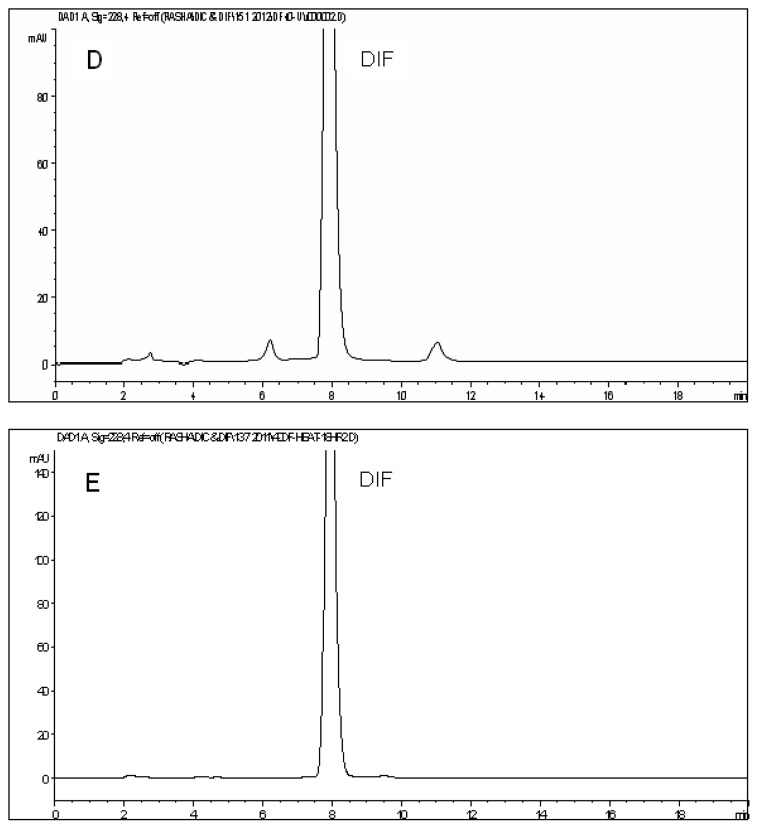
HPLC chromatograms of diflunisal after exposure to acid degradation (A), base degradation (B), oxidative degradation (C), photolytic UV degradation (D), and thermal dry heat degradation (E).

On the other hand, DIF was slightly susceptible to acid degradation. Heating of DIF with 1 M HCl solution at 90°C for 2 hr resulted in about 10% degradation with the emergence of three minor degradation peaks eluted at 10.99, 11.89, and 17.03 min (Fig. 6A). Similar to DIC, DIF appeared to be stable without any degradation in alkaline medium. Fig. 6B shows the peak of intact DIF after being heated at 90°C for 2 hr with 1 M NaOH. Oxidative degradation revealed an almost intact DIF peak with only 6% reduction in its peak area. Fig. 6C shows the chromatogram of DIF after treatment with 5% H_2_O_2_ at 90°C for 1 hr where no degradation peaks could be detected. Irradiation of DIF powder with UV light for 48 hrs caused about a 9% reduction in the peak area of the parent compound, in addition to the appearance of two new minor degradation peaks at 6.22 and 11.05 min (Fig. 6D). Finally, DIF was found to be stable under thermal (dry heat) degradation conditions. Its peak appeared at its specific retention time with area comparable to that of the standard of the same concentration, and the chromatogram did not show any extra peaks (Fig. 6E).

In all of these experiments, the resolution was calculated between either of the two analytes and the nearest degradation products peaks. Resolution was found to be no less than 2.01, which implies an adequate baseline separation between the two main compounds and any of the degradation products. It is noteworthy to mention that the peak purity test results obtained from the diode array detector (DAD) confirm that the DIC and DIF peaks are homogenous and pure in all of the analyzed samples subjected to forced degradation conditions.

## Conclusion

This study described a simple, selective, and reliable isocratic elution HPLC-DAD procedure for the assay of diclofenac sodium and diflunisal in their combined pharmaceutical dosage form. A significant advantage in this study is the separation of both analytes from the four different related substances, in addition to several degradation peaks obtained by hydrolytic, oxidative, and photolytic forced degradation experiments. The described method is superior to the previously reported analytical methods for the DIC-DIF mixture [34] since it is the first stability-indicating assay for this combination. Obviously, the described HPLC method offers a selectivity advantage over the spectrophotometric non-separation methods published in this previous report [34]. The diode array detector used in this study is in fact superior compared with the universal UV detector since the DAD has the advantage of being a tool for peak identity and purity confirmation. Reliability was guaranteed by testing the various validation parameters of the proposed method and successfully applying them to the commercial formulation. The method can thus be used for routine analysis, quality control, and for checking quality during stability studies of pharmaceutical preparations containing the two drugs.

## Figures and Tables

**Tab. 1:** Regression and statistical parameters for the determination of DIC and DIF using the proposed HPLC-DAD method.

Parameter	DIC	DIF
Concentration range (μg/mL)	5–100	5–100
Intercept (a)	−6.63	−15.88
S_a_	22.01	50.44
Slope (b)	44.32	120.33
S_b_	0.36	0.83
RSD% of the slope (S_b_%)	0.81	0.69
Correlation coefficient (r)	0.99987	0.99991
S_y/x_	28.27	64.82
F	14952	20963
Significance F	2.68 × 10^−8^	1.36 × 10^−8^
LOD (μg/mL)	0.65	0.26
LOQ (μg/mL)	2.16	0.87

**Tab. 2:** Precision and accuracy for the determination of DIC and DIF in bulk form using the proposed HPLC-DAD method.

Analyte	Nominal value (μg/mL)	Within-day			Between-day		
	
		Found ± SD^a^ (μg/mL)	RSD(%)	E_r_(%)	Found ± SD^a^ (μg/mL)	RSD(%)	E_r_(%)
DIC	20	19.86 ± 0.15	0.76	−0.70	19.92 ± 0.19	0.95	−0.40
	40	39.55 ± 0.22	0.56	−1.12	39.63 ± 0.34	0.86	−0.92
	80	80.13 ± 0.66	0.82	0.16	81.14 ± 0.93	1.15	1.43

DIF	20	19.91 ± 0.16	0.80	−0.45	19.84 ± 0.25	1.26	−0.80
	40	40.11 ± 0.25	0.62	0.28	39.83 ± 0.31	0.78	−0.42
	80	80.60 ± 0.54	0.67	0.75	81.23 ± 1.09	1.34	1.54

a Mean ± standard deviation for three determinations.

**Tab. 3:** Determination of DIC – DIF laboratory-prepared mixtures using the proposed HPLC-DAD method.

Nominal value (μg/mL)		Found ± SD^a^ (μg/mL)		RSD(%)		E_r_(%)	

DIC	DIF	DIC	DIF	DIC	DIF	DIC	DIF
20	80	20.24 ± 0.25	81.29 ± 0.75	1.24	0.92	1.20	1.61
20	40	19.85 ± 0.18	39.53 ± 0.34	0.91	0.86	−0.75	−1.18
40	40	39.79 ± 0.41	40.61 ± 0.39	1.03	0.96	−0.52	1.53
40	20	39.34 ± 0.31	19.82 ± 0.26	0.79	1.31	−1.65	−0.90
80	20	79.52 ± 0.70	20.19 ± 0.23	0.88	1.14	−0.60	0.95

a Mean ± standard deviation for five determinations.

**Tab. 4:** Analysis of DIC–DIF mixture in its pharmaceutical preparation (Rheumafen^®^ suppositories) by the proposed HPLC-DAD method and the reference method.

	External standard		Reference method [34]		Standard addition	
	
	DIC	DIF	DIC	DIF	DIC	DIF
%Recovery ± SD^a^	100.79 ± 1.11	100.97 ± 1.20	99.30 ± 1.44	100.24 ± 1.67	99.61 ± 1.38	101.16 ± 1.04
RSD%	1.14	1.19	1.45	1.67	1.39	1.03
t	1.83	1.93				
F	1.68	0.78				

a Mean ± standard deviation for five determinations.

Theoretical values for t and F at P = 0.05 are 2.31 and 6.39, respectively.
